# Supplementary material for: Deconvoluting simulated metagenomes: the performance of hard- and soft- clustering algorithms applied to metagenomic chromosome conformation capture (3C)
Source: PeerJ. 2016 Nov 8;4:e2676. doi: 10.7717/peerj.2676 (PMC5103821; doi:10.7717/peerj.2676)
Supplement: Table S1 [file peerj-04-2676-s001.pdf]

α_{BL}	Star			Ladder		
	ANI	1-ANI	$d_{*,*}$	$d_{A,[B\|C\|D]}$	$d_{B,[C\|D]}$	$d_{C,D}$
0.0250	0.9950	0.0050	<u>0.0050</u>	0.0075	<u>0.0050</u>	0.0025
0.0377	0.9930	0.0070	<u>0.0075</u>	0.0113	<u>0.0075</u>	0.0038
0.0568	0.9890	0.0110	<u>0.0114</u>	0.0170	<u>0.0114</u>	0.0057
0.0855	0.9830	0.0170	<u>0.0171</u>	0.0257	<u>0.0171</u>	0.0086
0.1288	0.9750	0.0250	<u>0.0258</u>	0.0387	<u>0.0258</u>	0.0129
0.1941	0.9630	0.0370	<u>0.0388</u>	0.0582	<u>0.0388</u>	0.0194
0.2924	0.9460	0.0540	<u>0.0585</u>	0.0877	<u>0.0585</u>	0.0292
0.4405	0.9230	0.0770	<u>0.0881</u>	0.1322	<u>0.0881</u>	0.0441
0.6637	0.8900	0.1100	<u>0.1327</u>	0.1991	<u>0.1327</u>	0.0664
1.0000	0.8470	0.1530	<u>0.2000</u>	0.3000	<u>0.2000</u>	0.1000
